# Association of Major Adverse Cardiac Events and Beta-Blockers in Patients with and without Atherosclerotic Cardiovascular Disease: Long-Term Follow-Up Results of the T-SPARCLE and T-PPARCLE Registry in Taiwan

**DOI:** 10.3390/jcm12062162

**Published:** 2023-03-10

**Authors:** Patrick Yan-Tyng Liu, Fang-Ju Lin, Chih-Fan Yeh, Yu-Chung Hsiao, Chin-Feng Hsuan, Wei-Tien Chang, Hsien-Li Kao, Jiann-Shing Jeng, Yen-Wen Wu, I-Chang Hsieh, Ching-Chang Fang, Kuo-Yang Wang, Kuan-Cheng Chang, Tsung-Hsien Lin, Wayne Huey-Herng Sheu, Yi-Heng Li, Wei-Hsian Yin, Hung-I Yeh, Jaw-Wen Chen, Chau-Chung Wu

**Affiliations:** 1 Division of Cardiology, Department of Internal Medicine, National Taiwan University Hospital, Taipei 10002, Taiwan; 2 Cardiovascular Center, Department of Internal Medicine (Cardiology), National Taiwan University Hospital, Taipei 10002, Taiwan; 3 Division of Cardiology, Department of Internal Medicine, Min-Sheng General Hospital, Taoyuan 33044, Taiwan; 4 Graduate Institute of Clinical Pharmacy, School of Pharmacy, College of Medicine, National Taiwan University, Taipei 10617, Taiwan; 5 Department of Pharmacy, National Taiwan University Hospital, Taipei 10002, Taiwan; 6 Division of Cardiology, Department of Internal Medicine, E-Da Hospital, Kaohsiung 82445, Taiwan; 7 School of Medicine, College of Medicine, I-Shou University, Kaohsiung 84001, Taiwan; 8 Department of Emergency Medicine, National Taiwan University Hospital, Taipei 10002, Taiwan; 9 Department of Neurology, National Taiwan University Hospital, Taipei 10002, Taiwan; 10 Division of Cardiology, Cardiovascular Medical Center, Far Eastern Memorial Hospital, New Taipei City 220, Taiwan; 11 Division of Cardiology, Department of Internal Medicine, Chang Gung Memorial Hospital, Taoyuan 333, Taiwan; 12 Division of Cardiology, Tainan Municipal Hospital, Tainan 701, Taiwan; 13 Division of Cardiology, Taichung Veterans General Hospital, Taichung 407219, Taiwan; 14 Division of Cardiology, Department of Internal Medicine, China Medical University Hospital, Taichung 404327, Taiwan; 15 Division of Cardiology, Department of Internal Medicine, Kaohsiung Medical University Hospital, Kaohsiung 80756, Taiwan; 16 Faculty of Medicine, College of Medicine, Kaohsiung Medical University, Kaohsiung 80708, Taiwan; 17 Division of Endocrinology and Metabolism, Department of Internal Medicine, Taipei Veterans General Hospital, Taipei 112201, Taiwan; 18 Division of Cardiology, Department of Internal Medicine, National Cheng Kung University Hospital, Tainan 704, Taiwan; 19 Division of Cardiology, Heart Center, Cheng-Hsin General Hospital, Taipei 112, Taiwan; 20 Departments of Internal Medicine and Medical Research, Mackay Memorial Hospital, Taipei 10449, Taiwan; 21 Department of Medical Research and Education, Taipei Veterans General Hospital, Taipei 112201, Taiwan; 22 Graduate Institute of Medical Education & Bioethics, College of Medicine, National Taiwan University, Taipei 10051, Taiwan

**Keywords:** beta-blocker, major adverse cardiac events, event-free survival, Taiwan cohort, atherosclerotic cardiovascular disease

## Abstract

Beta-blockers are widely used, but the benefit is now challenged in patients at risk of atherosclerotic cardiovascular disease (ASCVD) in the present coronary reperfusion era. We aimed to identify the risk factors of a major adverse cardiac event (MACE) and the long-term effect of beta-blockers in two large cohorts in Taiwan. Two prospective observational cohorts, including patients with known atherosclerosis cardiovascular disease (T-SPARCLE) and patients with at least one risk factor of ASCVD but without clinically evident ASCVD (T-PPARCLE), were conducted in Taiwan. The primary endpoint is the time of first occurrence of a MACE (cardiovascular death, nonfatal stroke, nonfatal myocardial infarction, and cardiac arrest with resuscitation). Between December 2009 and November 2014, with a median 2.4 years follow-up, 11,747 eligible patients (6921 and 4826 in T-SPARCLE and T-PPARCLE, respectively) were enrolled. Among them, 273 patients (2.3%) met the primary endpoint. With multivariate Cox PH model analysis, usage of beta-blocker was lower in patients with MACE (42.9% vs. 52.4%, *p* < 0.01). In patients with ASCVD, beta-blocker usage was associated with lower MACEs (hazard ratio 0.72; *p* < 0.001), but not in patients without ASCVD. The event-free survival of beta-blocker users remained higher during the follow-up period (*p* < 0.005) of ASCVD patients. In conclusion, in ASCVD patients, reduced MACE was associated with beta-blocker usage, and the effect was maintained during a six-year follow-up. Prescribing beta-blockers as secondary prevention is reasonable in the Taiwanese population.

## 1. Introduction

Risk factors predicting major adverse cardiac events (MACEs) are similar in patients with and without atherosclerotic cardiovascular disease (ASCVD) [1,2]. By controlling hypertension, hyperlipidemia, and diabetes mellitus, as well as lifestyle modification, physicians aim to lower the MACE occurrence. Several new medications are proven to lower MACE in primary and secondary prevention, such as sodium-glucose cotransporter 2 (SGLT2) inhibitors [3], glucagon-like peptide-1 (GLP1) receptor agonists [4], and statins, but some are shown non-beneficial. The role of beta-blockers is now under challenge.

Beta-blockers were recommended to lower mortality or cardiovascular events in patients with acute coronary syndrome (ACS) [5,6,7], silent ischemic heart disease [8], stroke [9,10], and peripheral artery disease (PAD) [8]. Since most studies were conducted in the pre-percutaneous coronary intervention era [11,12], newer cohort studies showed limitations in decreasing mortality in ACS patients without heart failure [13,14]. Long-term benefit in survival and cardiovascular events was also non-significant in post-MI patients in a three-year follow-up [15]. A meta-analysis published recently showed no association between beta blockers and all-cause mortality in the present coronary reperfusion era [16]. For primary prevention, the usage of beta-blocker was also suggested in some guidelines [17] since lower blood pressure per se could be beneficial to stroke prevention, heart failure, and CVD [8,18]. However, there were few studies targeting the association between composite MACE and beta-blockers in both primary and secondary prevention. We aimed to identify the risk factors of MACE and the long-term effect of beta-blockers in two large cohorts in Taiwan.

## 2. Methods

### Inclusion and Exclusion Criteria (the T-SPARCLE and T-PPARCLE Registry)

This study was conducted by the Taiwan Clinical Trial Consortium for Cardiovascular Diseases (TCTC-CVD), using the Taiwanese Secondary Prevention for patients with AtheRosCLErotic disease (T-SPARCLE) and Taiwanese Primary Prevention for AtheRosCLErotic disease (T-PPARCLE) Registry. The study design was published elsewhere [19]. Briefly, these two registries were initiated at 14 hospitals (eight medical centers and six regional hospitals) in order to recruit and follow a large population with or without atherosclerotic cardiovascular disease (ASCVD). These two cohorts included men and women aged >18 years. T-SPARCLE Registry enrolled the patients with evidence of ASCVD, which included (1) coronary artery disease (CAD, evidenced by cardiac catheterization examination, having a history of myocardial infarction, or with angina showing ischemic electrocardiogram changes or positive response to stress test); (2) cerebral vascular disease, cerebral infarction, intracerebral (excluding intracerebral hemorrhage associated with other diseases); (3) transient ischemic attack (TIA) with carotid artery ultrasound confirming atheromatous change with more than 70% blockage; or (4) peripheral atherosclerosis (symptoms of ischemia and confirmed by Doppler ultrasound or angiography). T-PPARCLE Registry enrolled the patients with no evidence of ASCVD but with at least one of the following risk factors: diabetes mellitus (DM), dyslipidemia, hypertension, chronic kidney disease (CKD), smoking, elder age (men > 45 years old, women > 55 years old), family history of premature CAD (men < 55 years old, women < 65 years old), and obesity (waist circumference: men > 90 cm, women > 80 cm). Patients were defined as having dyslipidemia if one of the following criteria was met: total cholesterol (TC) > 200 mg/dL; LDL-C > 130 mg/dL; TG > 200 mg/dL; men with HDL-C < 40 mg/dL or women with HDL-C < 50 mg/dL, or under lipid-lowering therapy. Hypertension and diabetes were diagnosed following conventional definitions and confirmed by the physicians that recruited the study participants. Chronic kidney disease was defined as patients with an estimated glomerular filtration rate (eGFR) < 60 mL/min/1.73 m^2^.

The exclusion criteria were patients with (1) other serious heart diseases; (2) ≥New York Heart Association functional class III heart failure; (3) life-threatening malignancy; (4) treatment with immunosuppressive agents; (5) other atherosclerotic vascular diseases with unknown disease type; (6) two or more statins treatment at enrollment; (7) chronic dialysis or (8) any condition or situation which, in the opinion of the investigators, might be not suitable for this registry study.

Eligible patients who fulfilled the enrollment criteria at the screening visit would be followed at six and twelve-month intervals and every year thereafter, and those with follow-up time <1 year but without MACE were also excluded. Written informed consent was obtained from each patient included in the study. The study protocol conformed to the ethical guidelines of the 1975 Declaration of Helsinki and was approved by the Taiwan Joint Institutional Review Board for each participating hospital (JIRB number 09-S-015).

## 3. Data Collection

The baseline characteristics, laboratory data and medication use were collected at the time of enrollment. During each follow-up, clinical endpoints, vital signs, concurrent medication, laboratory data, and other relevant clinical information were recorded. The patient’s demographic data, major vascular risk factors, previous disease history, and medications were collected according to a predetermined protocol. The body mass index (BMI) was calculated as the body weight divided by the square of the body height (kg/m^2^). Laboratory test results, including creatinine levels, total cholesterol (TC), triglyceride (TG), LDL-C and HDL-C, were obtained after enrollment. Non-high-density lipoprotein cholesterol (non-HDL-C) levels were calculated by subtracting the HDL-C from the total cholesterol (TC) levels. Serum creatinine was used to calculate eGFR by using the Modification of Diet in Renal Disease equation.

## 4. Outcome

The primary endpoint was defined as the time to the first MACE event after enrollment. MACE is a composite endpoint, including (1) cardiovascular death; (2) nonfatal stroke (ischemic stroke, hemorrhagic stroke, TIA or VBI); (3) nonfatal myocardial infarction (non-ST elevation acute coronary syndrome and ST-elevation myocardial infarction); and (4) cardiac arrest with resuscitation.

## 5. Statistical Analysis

Patients were classified into two groups: with and without ASCVD. The qualitative variable was summarized by count and percentage and compared with the chi-square test. For quantitative variables, the data were presented as mean ± standard deviation and analyzed with a student’s *t*-test. In order to identify independent risk factors on MACE occurrence, risk factors with a *p*-value less than 0.1 were included in the multivariate Cox proportional hazards model. Kaplan–Meier survival analysis was conducted separately in patients with and without ASCVD, and the difference was analyzed by log-rank test.

For missing data, we used multiple imputations (PROC MI procedure in SAS). The predictor variables in the imputation model include BMI, HDL-c, non-HDL, eGFR, heart failure and diabetes, as well as important non-missing variables such as age, gender, and MACE. In the study cohort, variables regarding the history of heart failure have missing values less than 5%; variables of BMI, HDL-c, non-HDL, eGFR and history of diabetes have missing values greater than 5%. The imputation step resulted in 20 complete data sets, each of which contains different estimates of the missing values for all 11,747 patients. After imputation, we used SAS/PROC PHREG to fit Cox proportional hazards (PH) model for each dataset and then used SAS/PROC MIANALYZE to combine results from the 20 Cox PH models.

## 6. Results

### 6.1. Risk Factors Associated with MACE in All Patients

A total of 12,224 patients were enrolled in the T-SPARCLE and T-PPARCLE registry between December 2009 and November 2014. After excluding patients with other serious heart disease or ≥ functional class III heart failure (n = 310), with dialysis (n = 131), and taking two statins (n = 17), 11,747 patients were included in the final analysis (6921 and 4826 in T-SPARCLE and T-PPARCLE, respectively). The median follow-up duration was 2.4 years, the same as the mean follow-up duration. Of all the enrolled patients, the mean age was 64.7 ± 11.8, and 62.8% were male. Among all the participants, 52.2% took beta-blockers.

During the follow-up period, 273 patients (2.3%) met the primary endpoint. These patients were older (64.6 ± 11.8 vs. 69.8 ± 12.3, *p* < 0.001), more likely to be male (62.7% vs. 68.9%, *p* < 0.05), with more smoking history (36.0% vs. 45.8%, *p* < 0.01), higher waist-hip ratio (0.92 ± 0.08 vs. 0.94 ± 0.08, *p* < 0.05), but lower body-mass-index (BMI) (26.3 ± 4.0 vs. 25.6 ± 4.3, *p* < 0.01). They were also with more history of CHF (8.2% vs. 20.5%, *p* < 0.001), DM (45.0% vs. 56.4%, *p* < 0.001), CAD (52.6% vs. 73.3%, *p* < 0.001), PAD (1.1% vs. 5.5%, *p* < 0.001), ischemic stroke or TIA (8.6% vs. 18.3%, *p* < 0.001), non-ischemic stroke (1.0% vs. 3.7%, *p* < 0.001), and CKD (23.1% vs. 47.8%, *p* < 0.001). More patients were on antiplatelet therapy (64.2% vs. 79.9%, *p* < 0.001), and fewer patients were on beta-blockers (52.4% vs. 42.9%, *p* < 0.01). The characteristics are summarized in Table 1. There was no significant difference in systolic blood pressure, lipid profile (total cholesterol, triglyceride, LDL, HDL, non-HDL-C), or history of hypertension.

### 6.2. Univariable Analysis of Risk Factors Associated with MACE

Among 6921 patients with ASCVD, several risk factors were identified for the occurrence of MACE in univariable analysis (Table 2), including older age (65.8 ± 11.6 vs. 69.9 ± 12.6, *p* < 0.001), lower BMI (26.3 ± 3.8 vs. 25.5 ± 4.1, *p* < 0.01), more history of CHF (10.6% vs. 22.0%, *p* < 0.001), DM (48.8% vs. 58.6%, *p* < 0.01), ischemic stroke or TIA (14.7% vs. 22.0%, *p* < 0.01), and CKD (26.3% vs. 48.3%, *p* < 0.001). These patients were less likely to take statins (66.5% vs. 59.0%, *p* < 0.05) and beta-blockers (54.8% vs. 41.9%, *p* < 0.001).

For 4826 patients without ASCVD, we found older age (62.9 ± 11.7 vs. 69.5 ± 10.7, *p* < 0.001), more history of CHF (4.9% vs. 13.0%, *p* < 0.05), and CKD (18.6% vs. 45.5%, *p* < 0.001) were associated with MACE occurrence (Table 2). These patients were more on antiplatelet therapy (35.7% vs. 54.4%, *p* < 0.05), but no difference was found in beta-blocker usage (49.2% vs. 47.8%, *p* = 0.88).

### 6.3. Multivariate Cox Proportional Hazards Model of Risk Factors Associated with MACE

With multivariate Cox PH model analysis (Table 3), the risk of MACE was significantly increased with age (Hazard ratio [HR] 1.01; 95% confidence interval [CI] 1.00–1.03), history of CHF (HR 2.14 [1.55–2.96]), and DM (HR 1.48 [1.12–1.95]) in ASCVD patients. The stage of CKD was positively associated with MACE (stage 4–5 vs. stage 1–2: HR 3.99 [2.47–6.44], stage 3 vs. stage 1–2: HR 1.73 [1.26–2.38]). Patients with low BMI had more MACE in comparison with normal or slightly overweight patients (HR 1.71 [1.23–2.36]). Increased non-HDL-c also predicted MACE occurrence, but the effect was only significant in higher levels (≥130 vs. <100: HR 1.48 [1.03–2.13]. Although statistically non-significant, there was a trend of decreased HR of the patients under statin therapy. Beta-blocker was the only independent protective factor (HR 0.72 [0.55–0.94]). In non-ASCVD patients, only age (HR 1.04 [1.01–1.06]) and the stage of CKD were also positively associated with MACE (stage 4–5 vs. stage 1–2: HR 5.24 [1.71–16.04], stage 3 vs. stage 1–2: HR 2.03 [1.04–3.94]) (Table 4). Although statistically non-significant, there was a trend of increased HR of the patients with a history of heart failure or under antiplatelet therapy.

### 6.4. Kaplan–Meier Survival Analysis of Long-Term Follow-Up

**Figure 1:**
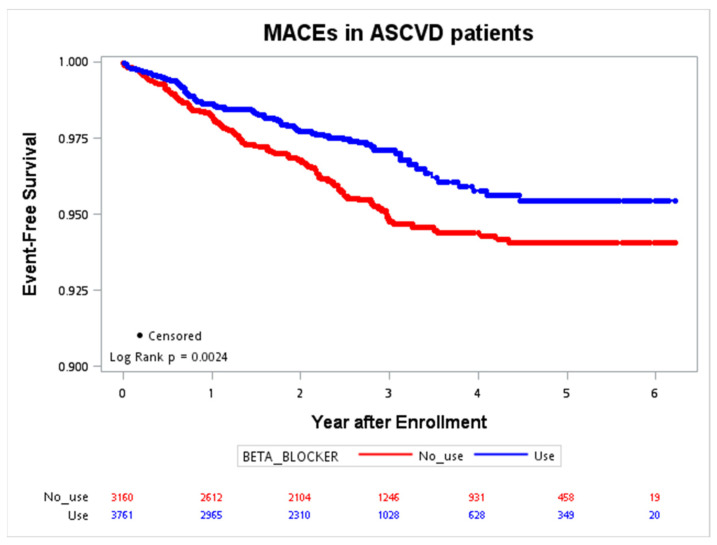
Event-free survival of the MACE (CV death, nonfatal stroke, nonfatal MI, cardiac arrest) in patients with ASCVD. ASCVD, atherosclerotic cardiovascular disease; CV, cardiovascular; MACE, major adverse cardiac event; MI, myocardial infarction.

**Figure 2:**
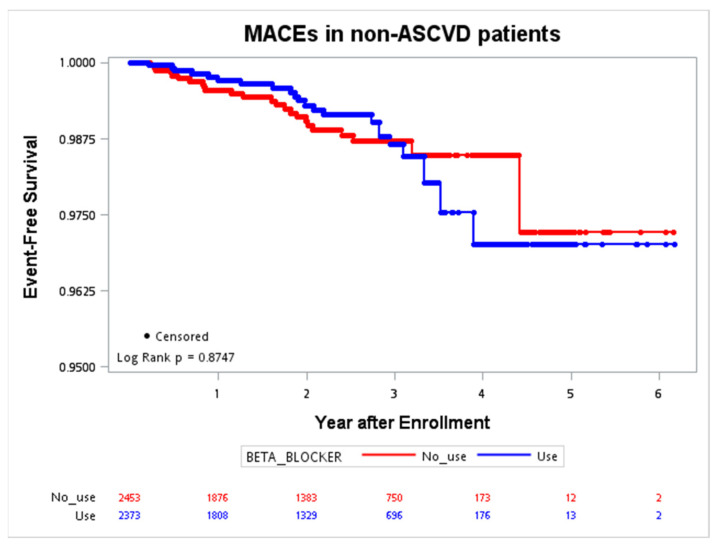
Event-free survival of the MACE (CV death, nonfatal stroke, nonfatal MI, cardiac arrest) in patients without ASCVD. ASCVD, atherosclerotic cardiovascular disease; CV, cardiovascular; MACE, major adverse cardiac event; MI, myocardial infarction.

The Kaplan–Meier curves for MACE occurrence in patients with and without ASCVD were plotted in Figure 1 and Figure 2. During the follow-up period in the ASCVD group, with a maximal interval of six years, the primary event rate was significantly lower in patients with beta-blocker use (*p* = 0.0024). The trend was not significant in patients without ASCVD (*p* = 0.8747).

## 7. Discussion

The T-SPARCLE and T-PPARCLE are two large registry cohorts initiated in several hospitals in order to analyze the MACE in patients with and without ASCVD in Taiwan. During a median 2.4-year follow-up, beta-blocker use was the only significant independent protective factor for MACE in secondary prevention, but it was not associated with a reduced MACE in primary prevention. It was also shown that the protective effect was maintained during the follow-up period. For both cohorts, elderly and CKD were associated with MACE, and additional independent risk factors, including low BMI, DM, CHF, and high non-HDL-c, were found only in ASCVD patients.

The role of beta-blockers in secondary prevention is controversial in the contemporary era, although it is still recommended in guidelines [5,7,17,20]. Since reperfusion therapy could limit the infarct size, the risks of recurrent MI, arrhythmia, and heart failure have declined [21]. For post-myocardial infarction patients without heart failure, some previous studies showed that prolonged beta-blocker treatment was not associated with reduced mortality [14,22]. In a propensity score-matched analysis from the Reduction of Atherothrombosis for Continued Health (REACH) registry, beta-blocker was not associated with a lower risk of MACE among patients with known prior MI, known CAD without MI, or with CAD risk factors [23]. Although lower mortality was observed in patients receiving beta-blockers within one year after MI in CLARIFY registry [14], the effect was not significant during further follow-ups up to five years. In contrast to these previous studies, the T-SPARCLE registry suggested fewer MACEs were associated with beta-blocker usage. The major difference between these registries was the study populations, of which Asians contributed to less than 20% of the REACH registry. In addition, the T-SPARCLE registry enrolled a relatively high percentage of patients with a history of myocardial infarction (74.8%) or diabetes mellitus (49.2%). Beta-blockers were known to be protective in ASCVD patients with diabetes [24]. Another registry done in Asian people with previous myocardial infarction, the Korea Acute Myocardial Infarction Registry-National Institutes of Health (KAMIR-NIH), also suggested a beneficial effect of beta-blocker use in the one-year risk of cardiac death [25]. The multivariable analysis of the T-SPARCLE registry showed that beta-blocker use was the only independent protective factor, instead of statin, ACEI or ARB, or antiplatelet therapy. It was also shown that a significant event-free survival remained during the follow-up period.

It is well known that beta-blockers could decrease oxygen demand due to reductions in heart rate, blood pressure, and contractility. It also prolonged the diastolic phase and increased coronary perfusion. Additionally, the antiarrhythmic effect and reduction of myocardial oxidative stress both contributed to its benefit [26]. Therefore, beta-blockers were recommended in the guidelines [5,7,17,20]. For patients with suspected CAD and stable angina symptoms, the benefit of beta-blocker has not been demonstrated in randomized controlled trials [2], but it is still recommended in current guidelines due to its anti-ischemic effect. In hypertensive patients, beta-blocker use has also been downgraded in current guidelines [8,17,18] since meta-analysis from previous studies failed to show its benefit in all-cause mortality, MI, or coronary heart disease [27,28]. In the T-PPARCLE population, this study showed beta-blocker use was not associated with decreased MACE in patients without ASCVD too. However, these hypertension treatment guidelines even impacted the physicians in Taiwan to under-prescribe beta-blockers for patients with ASCVD. This means this study could still show the protective effect of beta-blocker for ASCVD patients.

There might be several explanations for the neutral effect of statins, ACEI/ARB, and antiplatelet therapy for secondary prevention of MACE in the T-SPARCLE registry. In univariate analysis, those with MACE were less likely to take statins. Although statistically non-significant, there was a trend of decreased HR of the patients under statin therapy in multivariate analysis. These indicated statins still had a protective role. However, since the LDL and non-HDL levels had been low in the T-SPARCLE population, the protective effect of statin might be fading out.

Because ACEI/ARBs are independently associated with decreased mortality in all ischemic heart disease patients, current practice guidelines support the use of ACEI/ARB in patients with coronary artery disease without heart failure. However, a number of cited trials were performed prior to the era of prevalent statin use. When accounting for statin use in those without heart failure, the additive effects of ACEI/ARBs in reducing CV mortality appear to be nullified. Using data from the REACH registry, it has been shown that the use of ACEI/ARBs was not associated with better outcomes in stable CAD outpatients without HF [29]. A meta-analysis of ten ACEI trials and five ARB trials showed in CAD patients without HF, ACEI, but not ARBs decreased the risk of nonfatal MI, cardiovascular mortality and all-cause mortality, while both ACEI and ARBs decreased the risk of stroke [30]. In the T-SPARCLE registry, patients with advanced heart failure were excluded, and nearly 70% of the patients had received statin therapy. These might explain why there was no significant effect of ACEI/ARB on the MACE in the T-SPARCLE patients.

Antiplatelet therapy is also indicated for secondary prevention of MACE, and its neutral effect in this study might be due to the high prevalence (85%) of usage in ASCVD patients. In contrast, still more than one-third of T-PPARCLE patients were under antiplatelet therapy. Although statistically non-significant, there was a trend of increased HR of the patients under antiplatelet therapy. This implies that many of the clinicians in Taiwan still over-used antiplatelet medication in primary-prevention patients, although recently published guidelines did not recommend routine prescription of antiplatelets for primary prevention [31].

Several limitations of this study should be mentioned. This study was a prospective observational study in the Taiwanese population, so the finding is not as powerful as a randomized controlled trial, and the study population was limited to an Asian population sample. We did not analyze the kind and the dose of beta-blockers. For the enrolled people with ASCVD, CAD composed the main population (90%), followed by CVA (15%), while PAD contributed only 2%. Thus, the result might not reflect the patients of all kinds of ASCVD. There were also some limitations of the T-SPARCLE Registry, which were discussed elsewhere [24]. Mainly, the detailed history of each patient and the exclusion of patients with dialysis or advanced heart failure (NYHA Class III-IV) made extrapolation of the results beyond the study population with caution.

## 8. Conclusions

In conclusion, in ASCVD patients, beta-blockers were associated with a lower rate of MACE occurrence, and the effect remained in long-term follow-up even in the post-reperfusion and statin era. However, the benefit of beta-blockers was not significant in non-ASCVD patients. Prescribing beta-blockers for secondary prevention coincides with the recommendation in current guidelines.

## Figures and Tables

**Table 1:** Baseline characteristics of all patients by primary outcome events.

Variables, n (%)	Without MACE (n = 11,474)		With MACE (n = 273)		*p* Value
Age (yrs)	64.6 ± 11.8		69.8 ± 12.3		<0.001
Gender (Male)	7190	(62.7)	188	(68.9)	<0.05
Waist-hip ratio, mean ± SD	0.92 ± 0.08		0.94 ± 0.08		<0.05
BMI (kg/m^2^), mean ± SD	26.3 ± 4.0		25.6 ± 4.3		<0.01
Cigarette smoking history	4124	(36.0)	125	(45.8)	<0.01
Systolic BP, mean ± SD	133.3 ± 17.8		134.2 ± 18.9		0.16
History of HTN	8794	(76.8)	209	(76.6)	0.94
History of CHF	938	(8.2)	56	(20.5)	<0.001
History of DM	4784	(45.0)	149	(56.4)	<0.001
Previous coronary or LEAD intervention	3483	(30.4)	120	(44.0)	<0.001
CAD	6035	(52.6) *	200	(73.3) *	<0.001
h/o MI	5006	(43.6) *	174	(63.7) *	<0.001
PAD	126	(1.1)	15	(5.5)	<0.001
Ischemic stroke/TIA	983	(8.6) *	50	(18.3) *	<0.001
Non-ischemic stroke	117	(1.0) *	10	(3.7) *	<0.001
CKD (eGFR ≤ 60)	2389	(23.1)	120	(47.8)	<0.001
TC (mg/dL), mean ± SD	178.3 ± 39.7		176.8 ± 41.4		0.55
TG (mg/dL), mean ± SD	141.9 ± 97.0		145.4 ± 112.1		0.61
LDL (mg/dL), mean ± SD	103.5 ± 34.9		100.6 ± 35.6		0.20
HDL (mg/dL), mean ± SD	47.0 ± 14.1		46.3 ± 15.6		0.48
Non-HDL (mg/dL), mean ± SD	131.2 ± 38.2		130.4 ± 41.1		0.76
Statin use	6753	(58.9)	160	(58.6)	0.95
Fibrate use	687	(6.0)	11	(4.0)	0.20
With antiplatelet therapy	7367	(64.2)	218	(79.9)	<0.001
With ARB/ACEI	6497	(56.6)	160	(58.6)	0.54
With Beta-Blockers	6017	(52.4)	117	(42.9)	<0.01

* When assuming those with missing values didn’t have atherosclerotic vascular diseases. ACEI, angiotensin-converting-enzyme inhibitor; ARB, angiotensin II receptor blocker; BMI, body mass index; BP, blood pressure; CAD, coronary artery disease; CHF, congestive heart failure; CKD, chronic kidney disease; DM, diabetes mellitus; HDL, high-density lipoprotein; HTN, hypertension; LDL, lower-density lipoprotein; LEAD, lower extremity arterial disease; MACE, major adverse cardiac event; MI, myocardial infarction; PAD, peripheral artery disease; SD, standard deviation; TC, total cholesterol; TG, triglyceride; TIA, transient ischemic attack.

**Table 2:** Baseline characteristics of individuals by primary outcome events among patients with and without ASCVD.

	With ASCVD (n = 6921)			Without ASCVD (n = 4826)		
Variables, n (%)	Without MACE (n = 6694)	With MACE (n = 227)	*p*-Value	Without MACE (n = 4780)	With MACE (n = 46)	*p*-Value
Age (yrs)	65.8 ± 11.6	69.9 ± 12.6	<0.001	62.9 ± 11.7	69.5 ± 10.7	<0.001
Gender (Male)	4946 (73.9)	166 (73.1)	0.82	2244 (47.0)	22 (47.8)	1.0
Waist-hip ratio, mean ± SD	0.93 ± 0.08	0.94 ± 0.07	0.40	0.91 ± 0.08	0.92 ± 0.09	0.31
BMI (kg/m^2^), mean ± SD	26.3 ± 3.8	25.5 ± 4.1	<0.01	26.4 ± 4.2	26.0 ± 5.0	0.54
Cigarette smoking history	2952 (44.2)	110 (48.5)	0.22	1172 (24.6)	15 (32.6)	0.23
Systolic BP, mean ± SD	132.5 ± 18.1	134.0 ± 18.9	0.36	134.4 ± 17.3	135.3 ± 19.2	0.30
History of HTN	4810 (71.9)	168 (74.0)	0.55	3984 (83.6)	41 (89.1)	0.42
History of CHF	705 (10.6)	50 (22.0)	<0.001	233 (4.9)	6 (13.0)	<0.05
History of DM	3051 (48.8)	129 (58.6)	<0.01	1733 (39.5)	20 (45.5)	0.44
Previous coronary intervention	3483 (52.0)	120 (52.9)	0.84	N/A	N/A	N/A
CAD	6035 (90.2) *	200 (88.1) *	0.31	N/A	N/A	N/A
MI	5006 (74.8) *	174 (76.7) *	0.59	N/A	N/A	N/A
Ischemic stroke/TIA	983 (14.7) *	50 (22.0) *	<0.01	N/A	N/A	N/A
CKD (eGFR ≤ 60)	1576 (26.3)	100 (48.3)	<0.001	813 (18.6)	20 (45.5)	<0.001
TC (mg/dL), mean ± SD	170.3 ± 38.6	174.4 ± 41.8	0.12	189.4 ± 38.6	188.5 ± 37.6	0.88
TG (mg/dL), mean ± SD	140.0 ± 92.9	148.7 ± 119.5	0.29	144.5 ± 102.5	129.8 ± 65.6	0.14
LDL (mg/dL), mean ± SD	97.9 ± 33.9	99.7 ± 36.3	0.44	111.3 ± 34.7	105.2 ± 31.6	0.25
HDL (mg/dL), mean ± SD	45.0 ± 13.1	44.2 ± 12.7	0.40	49.7 ± 15.0	55.9 ± 22.8	0.08
Non-HDL (mg/dL), mean ± SD	124.8 ± 37.1	130.0 ± 42.2	0.09	139.8 ± 38.0	132.5 ± 36.1	0.20
Statin use	4453 (66.5)	134 (59.0)	<0.05	2300 (48.1)	26 (56.5)	0.30
Fibrate use	390 (5.8)	9 (4.0)	0.31	297 (6.2)	2 (4.4)	1.0
With antiplatelet therapy	5659 (84.5)	193 (85.0)	0.93	1708 (35.7)	25 (54.4)	<0.05
With ARB/ACEI	3839 (57.4)	136 (59.9)	0.45	2658 (55.6)	24 (52.2)	0.66
With Beta-Blockers	3666 (54.8)	95 (41.9)	<0.001	2351 (49.2)	22 (47.8)	0.88

* When assuming those with missing values didn’t have an atherosclerotic vascular disease; N/A: not applicable.

**Table 3:** Multivariate Cox PH model for predicting MACE among patients with ASCVD.

Parameters	Patients with ASCVD (n = 6921)			
	HR	HR 95% CI		*p*-Value
Age	1.01	1.00	1.03	0.04
BMI (vs. 23 ≤ BMI < 27.5 )				
BMI < 23	1.71	1.23	2.36	0.001
BMI ≥ 27.5	1.15	0.83	1.59	0.39
History of DM	1.48	1.12	1.95	0.01
History of CHF	2.14	1.55	2.96	<0.001
History of ischemic stroke/ TIA	1.15	0.83	1.59	0.40
Statin use	0.88	0.67	1.16	0.38
With beta-blockers	0.72	0.55	0.94	0.02
eGFR (vs. >60 mL/min)				
30 < eGFR ≤ 60 mL/min	1.73	1.26	2.38	0.001
eGFR ≤ 30 mL/min	3.99	2.47	6.44	<0.001
Non-HDL-c level (vs. <100 mg/dL)				
100 ≤ Non-HDL-c < 130	1.26	0.85	1.88	0.24
Non-HDL-c ≥ 130	1.48	1.03	2.13	0.03

**Table 4:** Multivariate Cox PH model for predicting MACE among patients without ASCVD.

Parameters	Patients without ASCVD (n = 4826)			
	HR	HR 95% CI		*p*-Value
Age	1.04	1.01	1.06	0.01
History of CHF	2.14	0.90	5.12	0.09
With antiplatelet therapy	1.46	0.81	2.63	0.21
eGFR (vs. > 60 mL/min)				
30 < eGFR ≤ 60 mL/min	2.03	1.04	3.94	0.04
eGFR ≤ 30 mL/min	5.24	1.71	16.04	0.004
Higher HDL-c level (vs. Lower HDL-c) *	1.19	0.63	2.27	0.59

* Low HDL-c was defined as <40 mg/dL for male and <50 mg/dL for female.

## Data Availability

Data available on request due to privacy and ethical restrictions.
